# Barriers and facilitators of HIV and hepatitis C care among people who inject drugs in Nairobi, Kenya: a qualitative study with peer educators

**DOI:** 10.1186/s12954-021-00580-7

**Published:** 2021-12-18

**Authors:** Natasha T. Ludwig-Barron, Brandon L. Guthrie, Loice Mbogo, David Bukusi, William Sinkele, Esther Gitau, Carey Farquhar, Aliza Monroe-Wise

**Affiliations:** 1 Department of Epidemiology, School of Public Health, University of Washington, 3980 15th Ave NE, Box # 351619, Seattle, WA 98195 USA; 2 Department of Global Health, School of Public Health and School of Medicine, University of Washington, 3980 15th Ave NE, Box #351620, Seattle, WA 98195 USA; 3 University of Washington Global Assistance Program-Kenya, Nairobi, Kenya; 4 Department of Medicine, Division of Allergy and Infectious Diseases, University of Washington, Box 356423, Seattle, WA 98195 USA; 5 VCT and HIV Prevention, Kenyatta National Hospital, Nairobi, Kenya; 6 Support for Addictions Prevention and Treatment in Africa (SAPTA), Nairobi, Kenya

**Keywords:** Kenya, Peer educators, People who inject drugs (PWID), HIV care, Hepatitis C care, Modified social ecological model (MSEM)

## Abstract

**Background:**

In Kenya, people who inject drugs (PWID) are disproportionately affected by HIV and hepatitis C (HCV) epidemics, including HIV-HCV coinfections; however, few have assessed factors affecting their access to and engagement in care through the lens of community-embedded, peer educators. This qualitative study leverages the personal and professional experiences of peer educators to help identify HIV and HCV barriers and facilitators to care among PWID in Nairobi, including resource recommendations to improve service uptake.

**Methods:**

We recruited peer educators from two harm reduction facilities in Nairobi, Kenya, using random and purposive sampling techniques. Semi-structured interviews explored circumstances surrounding HIV and HCV service access, prevention education and resource recommendations. A thematic analysis was conducted using the Modified Social Ecological Model (MSEM) as an underlying framework, with illustrative quotes highlighting emergent themes.

**Results:**

Twenty peer educators participated, including six women, with 2-months to 6-years of harm reduction service. Barriers to HIV and HCV care were organized by (a) individual-level themes including the competing needs of addiction and misinterpreted symptoms; (b) social network-level themes including social isolation and drug dealer interactions; (c) community-level themes including transportation, mental and rural healthcare services, and limited HCV resources; and (d) policy-level themes including nonintegrated health services, clinical administration, and law enforcement. Stigma, an overarching barrier, was highlighted throughout the MSEM. Facilitators to HIV and HCV care were comprised of (a) individual-level themes including concurrent care, personal reflections, and religious beliefs; (b) social network-level themes including community recommendations, navigation services, family commitment, and employer support; (c) community-level themes including quality services, peer support, and outreach; and (d) policy-level themes including integrated health services and medicalized approaches within law enforcement. Participant resource recommendations include (i) additional medical, social and ancillary support services, (ii) national strategies to address stigma and violence and (iii) HCV prevention education.

**Conclusions:**

Peer educators provided intimate knowledge of PWID barriers and facilitators to HIV and HCV care, described at each level of the MSEM, and should be given careful consideration when developing future initiatives. Recommendations emphasized policy and community-level interventions including educational campaigns and program suggestions to supplement existing HIV and HCV services.

## Introduction

In Kenya, people who inject drugs (PWID) are considered a key population that are disproportionately affected by the HIV and hepatitis C (HCV) epidemics, with prevalence estimates reaching upwards of 13–25% and 13–70%, respectively [1–6]. Within PWID communities, HIV-HCV co-infection varies by region, with prevalence estimates ranging from 5 to 32%, which is largely attributed to the coinciding transmission risks including unsafe injection practices (e.g., syringe sharing), sexual risks (e.g., unprotected sex, sexual violence, sex work), low HIV viral suppression (28–40%), and limited HCV prevention and care resources [1, 3, 7]. Moreover, PWID living with HIV are more susceptible to acquiring HCV and experiencing HCV-related morbidity and mortality [8]. Kenya’s national HIV program scale-up for key populations has resulted in significant reductions in HIV incidence among PWID, but service gaps remain with 57% of PWID unaware of their HIV status, 68% of PWID diagnosed with HIV are on antiretroviral treatment (ART), and 64% of those on ART are virally suppressed [4, 9]. With the release of direct acting antiretrovirals (DAAs), which are over 90% effective in treating HCV within other PWID settings, HCV elimination is achievable and has shown promising, cost-effective results when incorporated into existing harm reduction and HIV programs for PWID [9–12]. Within Kenya, DAA access has been largely cost-prohibitive, with few HCV prevention resources, and there is limited evidence on current PWID knowledge and perceptions of HCV, which can contribute to future HCV programs and services [1, 13]. As such, characterizing the circumstances surrounding PWID experiences of HIV and HCV care and access to prevention resources will provide opportunities to improve services uptake.

Kenya’s Ministry of Health supports the collaboration of healthcare providers and harm reduction organizations that have established rapport with PWID communities in order to address the HIV and HCV epidemics [14–16]. Harm reduction programs are largely credited with reducing HIV and HCV incidence among PWID through needle-syringe programs (NSPs), HIV counseling and testing services, social services (e.g., meals, shower facilities) and healthcare referrals [9, 17]. Common healthcare referrals include HIV and HCV care, wound care and opioid substitution therapy (OST), with several OST clinics providing more comprehensive healthcare services by offering both OST and HIV treatment to PWID [18]. Currently, OST offers directly observed methadone treatment, which requires patients to visit clinic locations daily, creating barriers around transportation, household responsibilities and financial hardship [18, 19]. Despite the success of harm reduction programs, NSP and OST service coverage remains low with approximately 54% of PWID accessing NSPs and 4% enrolled in OST [20]. In Nairobi, Support for Addictions Prevention and Treatment in Africa (SAPTA) is a non-profit organization that implements an evidence-based Peer Educator (PE) Program that trains former PWID to conduct outreach in PWID communities, offering grassroots harm reduction services that reduce HIV and HCV incidence, and provide assisted referrals for clinical services [21]. PEs serve as trusted community liaisons, often bridging the gap between PWID and healthcare providers, who largely serve the general public. Until recently, most studies have highlighted the experiences and perspectives of PWID, healthcare service providers and key stakeholders, but rarely consider the unique vantage-point of PEs, who offer both personal and professional perspectives on dynamics surrounding addiction, HIV and HCV care, and recommendations for improving service uptake [13, 19].

The Modified Social Ecological Model (MSEM) provides a framework for understanding the multiple contributors to infectious disease risk within specific communities, but it has yet to be applied to PWID communities within Kenya [22]. The MSEM suggests that individual behaviors are not solely derived from individual decisions, but rather influenced by external factors outside of an individual’s control [22, 23]. Specifically, the MSEM explains the complex relationships between the stage of an epidemic, including HIV and HCV epidemics, and the surrounding risks that fall within political, community, social network and individual domains [22, 23]. Policy-level risks include laws and policies that promote or prohibit access to care, whereas community-level risks include organizational structures, social cohesion, and socio-cultural norms that may affect a PWID’s ability to engage in HIV and HCV care [22]. At the social network-level, risks may be influenced by family, friends, sexual and/or injecting partnerships, while individual-level risks include personal knowledge, attitudes, and behaviors that influence the personal health and wellbeing of PWID [22]. Moreover, the MSEM applies an ecological approach to understanding health outcomes so that individuals are able to influence, and be influenced by, their social networks, community and policies that govern those communities, which come together to impact HIV/HCV service uptake. MSEM levels are not independently operationalized, but often contain dynamic, mutually reinforcing relationships that influence one another and may change over time [22, 23]. Describing HIV and HCV barriers and facilitators to care through an ecological lens, applying the MSEM, will provide a holistic view of how the surrounding environment impacts PWID behaviors.

Using the MSEM as an underlying framework, we aim to characterize barriers and facilitators of HIV and HCV care through the perspective of community-embedded PEs in Nairobi, Kenya. Until recently, several studies have highlighted the roles and perspectives of clinicians, policy makers and current PWID, but rarely seek input from PEs who offer intimate knowledge of PWID experiences. In addition, we ask PEs for suggestions on resources and services that may support current PWID and aide their work in improving HIV and HCV care uptake, with the ultimate goal of achieving community-level HIV viral suppression and HCV viral clearance.

## Methods

### Setting

Our qualitative study takes place within Nairobi’s urban core with two SAPTA facilities where PEs provide outreach and harm reduction services to surrounding PWID communities. The SAPTA service region caters to roughly 5,000–11,000 PWID, most of whom buy, sell and trade drugs within “dens” that are located in outdoor public spaces [9]. To avoid contributing to community stigma, location names will not be provided. PEs are trained harm reduction specialists, with a history of substance use disorder and many are former PWID. They undergo instructional and field training in order to provide a range of harm reduction services, including distributing NSP kits; connecting PWID to HIV/HCV counseling and testing services; educating on HIV, HCV and overdose prevention; providing opioid-receptor agonist treatment (i.e., naloxone); promoting OST clinical referrals; providing medical navigation assistance (e.g., appointment setting and reminders, transportation); and encouraging the use of social services offered through SAPTA facilities (e.g., meals, groceries, shower and laundry facilities) At the time of this study, HIV treatment and care was subsidized throughout Nairobi; however, HCV treatment was limited to interferon-based regimes, that were largely cost prohibitive and < 50% effective [1]. Until recently, DAA treatment was limited to research studies and pilot programs, with a national HCV treatment rollout anticipated in 2021. Strategic planning around HCV services and DAA dissemination are expected to evolve in order to meet community needs.

### Sampling and recruitment

We established a sampling frame of 60 PEs working at two SAPTA facilities in Nairobi, Kenya. Study eligibility included: (1) adults 18 years or older; (2) employed as a PE in September 2017; (3) English or Swahili-speaking; and (4) willing and able to provide informed consent. All SAPTA PEs are former persons who use drugs, with the majority being PWID (> 90%), and are considered trusted community liaisons. Random and purposive sampling techniques were applied, whereby we randomly selected PEs from a roster and oversampled female PEs in order to increase the robustness of participant experiences. While women who inject drugs are a minority within PWID communities in Kenya, recent evidence indicates that women may experience unique social, structural, economic and political barriers and facilitators to accessing HIV/HCV care, compared to their male counterparts [24, 25]. By oversampling PEs who are women, we aim to capture a wide range of experiences including poor treatment by clinical personnel, policing practices, stigma, household responsibilities, and other factors that may not be experienced by men. A standard script was used to explain the study purpose and procedures, and in total, 20 participants were selected and agreed to participate.

### Data collection and management

During the study’s formative phase, key informant interviews were completed by the senior author (AMW), who met with SAPTA leadership, HIV and HCV clinicians, mental health experts, government officials, and community activists, which helped inform the research aims and interview guide questions. A priori research questions included HIV barriers and facilitators to care, with the inclusion of HCV-related aims based on key informant suggestions. The interview guide was developed by several co-authors, including SAPTA leadership, through an iterative process. The guide was translated into the local language (Swahili) by the study coordinator (LM), who is a native Kenyan, bilingual (English and Swahili) and has extensive experience working with PWID living with HIV. Review and back-translation of the guide was completed by a SAPTA manager (EG), who is bilingual and bicultural. Prior to the start of data collection, the interview guide was pilot tested with two PEs to ensure colloquial phrases commonly used between PEs and clients were incorporated and applied appropriately.

Semi-structured, in-depth interviews were used to elicit PEs personal and professional experiences surrounding (1) job responsibilities; (2) PWID access and utilization of HIV, HCV and addiction care services; (3) treatment by law enforcement and medical providers; and (4) suggested resources for PWID and PEs who provide harm reduction services. Data collection occurred from September to December 2017. One female, bilingual (English/Swahili) interviewer, with graduate-level qualitative training and extensive experience working with local PWID communities, received training on study procedures and conducted all interviews. Interviews were audio recorded and ranged from 45 to 90 minutes, with the interviewer taking detailed field notes to summarize the interview content and the physical and mental condition of each participant. Weekly study team discussions were used to (a) refine the interview guide, (b) explore emergent topics (i.e., reciprocation), and (c) initiate analysis discussions [26–28]. Through study discussions, research team members concluded conceptual saturation had been reached, whereby additional interviews would not elicit new information [26, 27]. All transcripts were transcribed verbatim, with all but four transcripts undergoing translation from Swahili to English. ATLAS.ti, v8 (Berlin, Germany), was used to manage and analyze all transcripts, field notes, and memos into one integrated system.

### Data analysis

We conducted a thematic analysis using similar methods to those described by Braun and Clark (2006) [26]. Codebook development involved three study team members (NLB, AMW and LM) independently reading and open-coding select transcript excerpts in order to generate an initial list of codes based on a priori topics (i.e., deductive) and emergent themes (i.e., inductive) [26, 28]. Similar themes were merged together as common or recurring concepts, which were organized into typologies and later into classification schemes. Weekly coding schedules consisted of coding, reviewing field notes, and writing detailed memos, which was followed by study team discussions of major themes and code definitions as an iterative process [28]. Isolated coding concerns were resolved through team discussions and further refinement of codebook parameters. Higher-level code classifications included (a) drug use and addiction, (b) social support systems, (c) politics and law enforcement, (d) infectious diseases, and (e) peer educator employment. Guided by the MSEM, PWID experiences of HIV/HCV barriers and facilitators to care, as described by PEs, are presented alongside representative quotes using pseudonyms to protect participant anonymity.

### Ethical approval

All study procedures and materials were approved by the University of Washington Institutional Review Board (Seattle, WA, USA) and the Kenyatta National Hospital/University of Nairobi Ethical Review Committee (Nairobi, Kenya). Participants provided written informed consent in Swahili or English. Careful consideration was given to SAPTA employment contracts, which outlines grounds for probation or dismissal if a PE disclosed current substance use, particularly the combination of OST and substance use. Whether PEs admitted to current drug use or felt that the interview content would trigger the use of substances post-interview, they were encouraged to speak with an addiction counsellor. To our knowledge, no PEs were engaging in substance use or felt the need to use substances at the time of their interview, and none were dismissed or placed on probation. PEs were reimbursed 400 Ksh ($4 USD) for their time and transportation.

## Results

Twenty participants completed in-depth interviews, of which 30% were women. PE mean age was 37 years, half completed their secondary education (55%), and more than half were married or had partners (65%). Participant average years of service was 3 years (range: 2 months-6 years) (Table 1). PEs referred to PWID living with HIV/HCV as their “clients,” which is how they will be referred to throughout.

**Table 1:** Study population characteristics

Characteristics	Peer educators (n = 20)
Sex	
Female Male	6 (30%) 14 (70%)
Mean age (range)	37 years (25–50 years)
Level of education	
Primary Secondary Some college	9 (45%) 10 (50%) 1 (5%)
Married or partnered	13 (65%)
PE average years of service (range)	3 years (2 months–6 years)

**Fig. 1:**
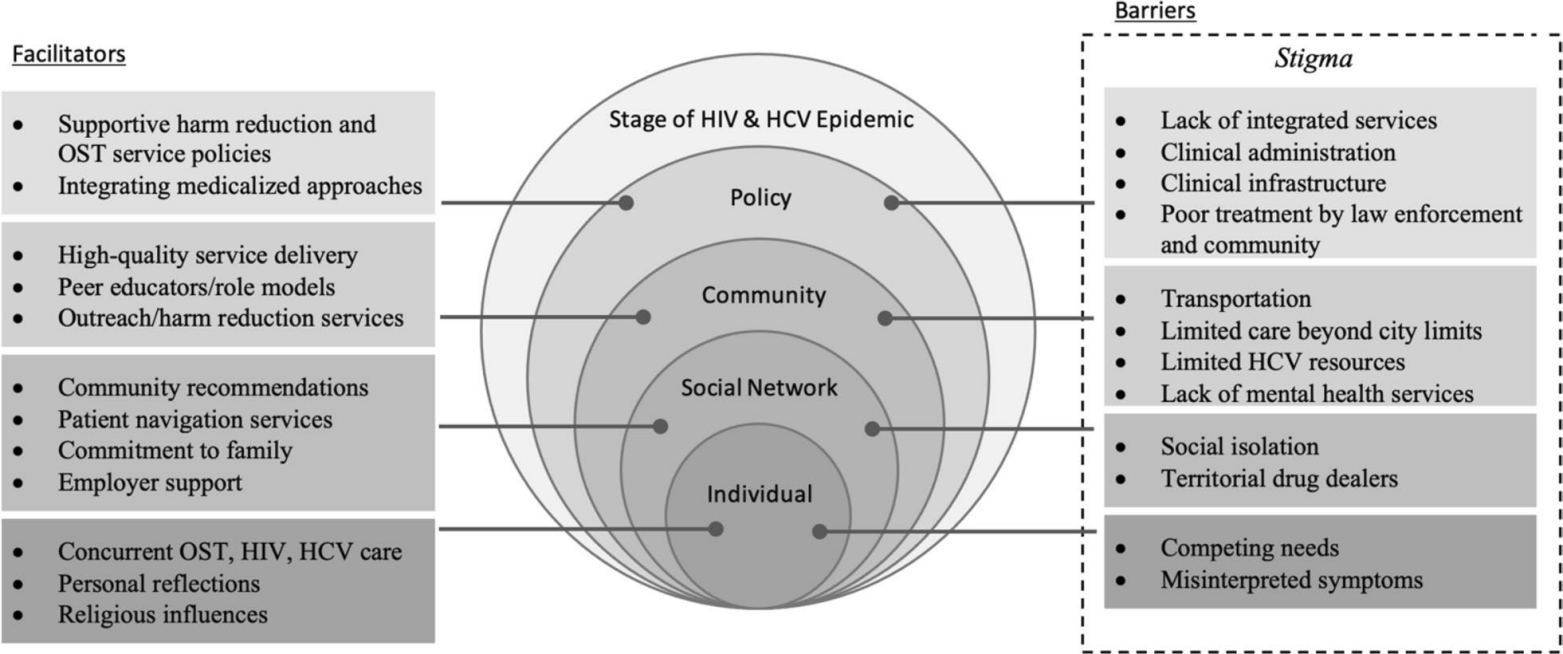
Summary of HIV/HCV care themes applied within the Modified Social Ecological Model (MSEM)

Themes relevant to HIV/HCV barriers and facilitators to care were adapted to the MSEM framework including individual, social network, community and policy-level factors that are centered around Kenya’s HIV and HCV epidemics (Fig. 1), with suggested service recommendations that included medical, social and ancillary support services, provided at the end.

### Barriers to HIV and HCV Care

#### Individual-level themes

Participants described two individual-level barriers: the competing needs of addiction and misinterpreted HIV/HCV symptoms. PEs characterized periods of addiction, from both personal and professional perspectives, as constantly having to “hustle” to earn an income to acquire drugs, food and shelter, with a daily objective to avoid withdrawal symptoms. As a consequence, HIV/HCV treatment and care was often neglected. Kizito, a PE for over 2 years, described the effort he dedicates to escorting clients to HIV/HCV testing and treatment facilities, but struggles to keep his clients engaged in care when clients anticipate their withdrawal symptoms in-transit or waiting in queue to see a provider at the clinic. He described one client with HCV complications, who voluntarily discharged against medical advice:

*I lost one guy with hepatitis C and the problem is that they [PWID clients] cannot be contained. They don’t stay in the hospital. If they are admitted, when they feel a little bit okay, they run away because of the craving. *Kizito, M, age 44 years, PE for 2 years

Kizito spends a large portion of his day trying to overcome his clients’ competing needs of addiction, particularly when transporting clients to multiple medical facilitates or while in waiting queues. He concludes that without PE assistance and follow-up, clients will delay their HIV/HCV medical care until they are extremely sick and treatment options are limited.

In other cases, PEs describe their clients’ hesitation in receiving an HIV/HCV test due to their misinterpretation of *rosto*, or withdrawal symptoms, which can be difficult to discern from symptoms of advanced HIV/HCV infection (e.g., headaches, body chills, stomach aches, diarrhea, etc.) Below Murwa, a newly trained PE, explains that some of his clients have mistaken HIV/HCV-related health issues as withdrawal symptoms, causing them to consume additional drugs rather than seek care:

*You might find that someone has stayed with the HIV virus for almost one-year and they don’t know [their serostatus], and the body continues to wither down.. . .they feel the stomach or somewhere else aching, and they will just say it is rosto [withdrawal symptoms]. *Murwa, M, 37 years, PE for 7 months

Experiences of HIV/HCV-related health issues could be the result of high viral loads, stemming from not taking treatment. When paired with sharing injection equipment or unprotected sexual activities, PEs highlighted that their clients risk transmitting HIV/HCV to sexual and injecting partners.

#### Social network-level barriers

PEs highlighted two social network-level themes: social isolation and territorial drug dealers. For many clients, stealing from family and friends is a common occurrence, with all but two PE’s recalling personal and professional accounts of theft that resulted in dissolved relationships. Otieno (M, 36 years), a PE who injected heroin for 15 years, recalled experiencing social isolation from family and friends following his HIV diagnosis that impacted his mental health. He relates to clients experiencing isolation and fatalistic thoughts where, “*they don’t see the meaning of life*”, which ultimately affects their willingness to engage in HIV/HCV care. In addition, PEs described limited family counselling services that are tailored to PWID, PWID living with HIV and their families. More than half of the PEs described the benefits of addressing clients’ mental health and supporting family reunification, which could increase engagement in HIV/HCV services.

Territorial drug dealers, interfered with PEs’ ability to provide harm reduction services in local dens, or outdoor areas where drugs are bought, sold and consumed. Moha describes acts of physical violence and threats from drug suppliers, who blame PEs for impinging on their revenue:

*Sometimes you have to hide, so that you can talk with people [clients] at the den, or when distributing the [needle-syringe] kits, sometimes we hide because the drug lords see us as blocks to their business. . . . we [PEs] are people who have been marked. There was a time a peer educator from here, they were beaten.* Moha, M, 34 years, PE for 2 years

PEs provide essential harm reduction and HIV/HCV services, which is obstructed when drug dealers threaten or carry out acts of violence. As a result, clients will avoid PEs, fearing retaliation by drug dealers, which limits their access to harm reduction services.

#### Community-level barriers

Four community-level themes emerged during PE discussions including: (a) transportation, (b) limited care beyond city limits, (c) limited HCV resources, and (d) lack of mental health services. PEs described transportation cost as a common barrier, especially when clients lived or spent time outside of city limits. Three PE’s, including Wangai, described instances where they felt obligated to fund transportation costs to medical facilities when their client’s health was at stake:

*If the person is very sick, they will have to use transport and you find that we don’t have money in the DIC [SAPTA drop-in centre] to support such things. We contribute money amongst ourselves [between several PEs] and take him to the hospital. *Wangai, M, 42 years, PE for 5 years

Wangai and Muniu (M, age 39) explained that their personal experience with addiction motivates them to support clients who are “still our brothers and sisters”, regardless of the financial cost. On several occasions, PEs described selfless acts to overcome community-level barriers, which highlights their dedication to client’s healthcare needs and goes beyond financial incentives.

In addition, PEs described several scenarios where clients with HIV/HCV-related complications returned to their home villages to receive family support. However, with limited medical facilities in the villages, family members will often transport clients back to Nairobi when their health conditions are severe. To complicate matters, clients often do not disclose their HIV/HCV-status or substance use to family members, due to stigma and fear of discrimination. Rita and her sister moved into a Nairobi slum in search of work, where they both developed a heroin addiction. Rita received her HIV diagnosis shortly after her sister passed away, and although her sister was never tested, Rita assumes her death was related to HIV complications. Rita summarizes her sister’s final days alive:

*She [Rita’s sister]got pneumonia. . . I took her home [to our village] when I saw things are bad and by that time she was really in a bad condition. My mother took her to the hospital, and the following day when my mother went to visit her, found that she had died. You know when you are at the base. . . you don’t think that this thing is serious.* Rita, F, 38 years, PE for 3 years

Rita was one of two PEs that described returning home to their village for family support and finding limited medical resources. In addition, many clients underestimate the severity of their illness and re-engage in care when they are severely immunocompromised, which limits treatment options and increases the risk of mortality.

Several PEs cited an abundance of HIV-related health education and awareness, but limited community awareness on HCV testing, treatment and care options. Muniu (M, 39 years) a PE for more than 4 years, describes his clients’ limited awareness of HCV resources and requested national HCV educational campaigns to educate PWID communities on transmission, affected sub-groups, and locations for low-cost testing services. In addition, Muniu and one other PE discussed recent public health campaigns around hepatitis B, which created confusion among their clients, who find the similar nomenclature to be challenging when trying to distinguish between the two infectious diseases. While there has been consistent community-level HIV prevention messaging promoting the use of clean injection equipment and condoms, clients often lack information on HCV prevention services and treatment options.

Nearly all PEs described personal and/or professional experiences of poor mental health that impacted daily activities, drug use, and ultimately, HIV/HCV care. Descriptions of violence, trauma, stress, isolation and experiences of forced migration to major cities in order to support family members, all contributed to poor mental health. Seventeen PEs described experiences of trauma, stress, depression and anxiety after witnessing a peer overdose. Muniu (M, age 39 years) recalled a traumatic event where he and his peers attended a demonstration event that turned violent and his close friend was killed, citing, “*Nothing like that has ever happened to me. Losing someone that you were with five minutes ago, I think it took a toll on me*”. Following the event, Muniu’s addiction to heroin and benzodiazepines increased and he neglected his HIV care for more than a year. While PEs draw on personal experiences to counsel clients, they recognize their limitations in providing professional mental health services. Furthermore, PEs conveyed that most mental health services do not treat co-occurring disorders or severe mental health conditions (e.g., suicidal ideation, depression, psychosis), but highlighted the importance of mental health services and improved HIV/HCV care.

#### Policy-level barriers

PEs described four policy-level themes: integrated and tailored services, clinical administration (e.g., long wait queues, ambiguous financial obligations), clinical infrastructure that creates confidentiality concerns, and poor treatment by law enforcement and community members. PEs consistently cited the lack of integrated, tailored services as reasons for discontinued HIV/HCV care among their clients. Many endorsed a “one-stop” facility where clients could access harm reduction, HIV/HCV care, OST, wound care, mental health and ancillary services from providers who understood the specific needs of their clients. Harm reduction sites are limited to basic first aid, HIV counseling and testing, peer support groups and social services, with all other medical services provided via assisted referrals. Patient referrals to HIV/HCV care and OST services are coordinated by SAPTA staff, including PEs, to help overcome the barriers of scheduling, transportation and locating facilities equipped to assist PWID. OST services, HIV and HCV care are typically offered in separate clinic locations, with the exception of OST clinics that recently began offering daily OST and ART. As a result, clients that attend multiple clinics face additional time and financial burdens associated with lost income, transportation costs and household obligations. Nearly all PEs advocated for integrated medical services provided within harm reduction facilities, which have established positive report with PWID.

Administrative barriers included crowded medical facilities, long wait-queues and ambiguity around the financial obligations for medical services. In several cases, PEs felt obligated to accompany their clients to medical visits in order to provide coping strategies for withdrawal symptoms and to assist in navigating complex administrative processes. In addition, ambiguous financial obligations leave PWID unsure about whether medical expenses are subsidized or unsubsidized, creating unnecessary barriers to HIV/HCV and ancillary health services. Kizito described accompanying his clients to medical appointments and being solicited for payment:

*Once we take the client to the hospital, we take the client to the Social Department where they [apply for a subsidization] waiver. It is a challenge because if they [medical staff] refuse, you keep on telling them that they are street people and they are junkies and we usually have letters with our [SAPTA] letterhead, used for referrals. And then, at long last, they agree to waiver. That is why you have to follow up with them . . because if you leave them [a client] there, they will just die.* Kizito, M, age 44 years, PE for 2 years

While HIV medical services are subsidized, clinical administrators will often seek payment for ancillary medical services, including abrasions, infections and bone fractures, that can delay PWID from seeing a provider and increases the possibility of experiencing withdrawal symptoms and undue stress. Like Kizito, many PEs feel obligated to assist clients in navigating through administrative and clinical barriers, fearing clients will become overwhelmed and neglect their medical care.

Several PEs highlighted concerns surrounding confidentiality breaches due to the lack of privacy within the clinical infrastructure. Often small building spaces and the use of curtains as temporary walls impacted privacy, so that patients in the waiting areas could decipher patient-provider conversations. Kiplimo understands the advantages of providing ART within OST clinics, but described his clients’ confidentiality concerns:

*You will be given your medicine [ART] and then you are given your methadone [OST] through the window and you know there is no curtain. . . . Or even there are two windows, and you open the other one and you find someone receiving the medicine and then that person will start talking.* Kiplimo, M, 35 years, PE for 6 years

Kiplimo and two other PEs described the benefits and convenience of integrating ART and OST services; however, breaches in confidentiality have led to the unintended disclosure of clients’ HIV status, which later induced social discrimination. The fear surrounding confidentiality breaches causes clients to avoid OST clinics or to enroll in separate facilities. Notably, clients that attend separate medical facilities face both time and financial constraints, which can increase suboptimal care through missed appointments and treatment regimens.

Nineteen PEs described first- and second-hand accounts of poor treatment, harassment, and violent acts that were carried out by local businesses, police and community members. Fifteen PEs described personal and secondhand experiences of physical violence perpetrated by community members, referred to as “mob justice”, with at least three instances resulting in mortality. Mob justice was described as a spontaneous assembly of community vigilantes delivering justice through physical beatings, typically following theft or the destruction of property. In addition, 13 PEs characterized scare tactics, threats and acts of physical violence carried out by law enforcement. Kizito (M, 44 years) described one law officer who consistently carried out lethal force within the dens, which resulted in more than a half-dozen deaths. According to Muniu (M, age 39 years), law enforcement agents arbitrarily apply physical violence towards PWID, explaining, “*...there [are] good days and bad days for a cop*”, which causes clients to avoid areas associated with law enforcement and government entities, including medical facilities that provide HIV/HCV services. While law enforcement agents avoid the areas surrounding harm reduction facilities, there is a high likelihood of encountering law enforcement agents in transit or around HIV/HCV clinics, which is a concern for PWID. Law enforcement mistrust was rooted in corruption, with three PEs describing personal bribe solicitations by officers in order to mitigate jail sentences. This was coupled with recent government sanctioned den raids carried out by law enforcement, where surrounding homes were demolished within hours and physical violence targeted known PWID. Raids caused displacement, stress, and trauma amongst clients, with several PEs unable to locate clients for medical appointments. Considered together, displacement, corruption and violence perpetrated by law enforcement and supported by government officials, further marginalize PWID and limited their access to HIV/HCV healthcare services.

#### Overarching barrier: stigma

Discussions around stigma-related barriers to HIV/HCV care were described at each level of the MSEM. PEs described intrapersonal stigma as clients’ acceptance of discriminatory beliefs about themself that were rooted in living with HIV/HCV, using drugs, and/or illegal income sources (e.g., “hustling”, stealing, sex work). At the social network-level, PEs frequently described experiences of stigma, isolation and being outcasted from friends, family and community members based on one’s addiction, illegal income sources, and/or HIV/HCV serostatus status. Cherono (F, 33 years) still faces the consequences of her heroin addiction, as her siblings are unwilling to communicate. As a PE, Cherono witnesses similar patterns of family isolation stating, “*They [family] count a junky as dead, they don’t count them as a person anymore*”. At the community-level, PEs highlighted experiences of discrimination by uninformed medical providers and law enforcement, which further isolates PWID. Rita (F, 38 years) and two other female PEs explained that doctors that serve *raiya*, or the general public, “*look at you like you are not human*”, which further marginalizes PWID. Often PEs characterized societal beliefs associated with substance use and HIV/HCV as lapses in judgement or poor morality, which causes clients to avoid medical assistance. Experiences of clinical stigma and discrimination caused several clients to go without screenings, care, and treatment. Particularly, six PEs described medical conditions (e.g., abscesses, broken bones and high fevers) that were left unattended and increased in severity, which complicated treatment options. At the policy-level, PEs discussed historically unsupported harm reduction programs by government officials that marginalized PWID, which was compared with the current lack of government support for HCV services. In general, PEs perceived policy-makers, government officials, medical providers, and community members as being uninformed and intolerant towards their clients’ needs.

### Facilitators to HIV and HCV care

#### Individual-level facilitators

PEs characterized three individual-level themes, including: concurrent engagement in OST, personal reflections and religious influences that motivated clients to remain engaged or re-engage in HIV/HCV medical care. Almost all PEs described improvements in physical and mental health when HIV, HCV and substance use disorders were addressed in parallel. Particularly, OST clinics have integrated ART within their services, with infrastructure to accommodate HCV testing and DAA distribution in the future. In addition, several PEs discussed personal reflections following severe health complications or the death of a peer, as a motivating factor for returning to HIV care. The fear of death, particularly not wanting to abandon family or children, prompted several PEs to prioritize their health, re-engage in care and reduce their substance use. PEs credit their lived experiences and personal reflections as impactful tools in encouraging clients to engage in care. Furthermore, 10 PEs credited their faith and religious beliefs for improvements in treatment adherence and overall health, especially following extended periods of sub-optimal care. Cherono (F, age 33 years) reflected on surviving a 9-year heroin addiction, being orphaned, surviving physical violence, and living in unsafe environments stating, “*I don’t have parents, no one else to take care of me, only God*.” Cherono shares with clients that her strength and survival is a combination of religious faith and following medical advice, which has allowed her to be a better mother. Several PEs described the benefits of faith-based organizations that provide social services (e.g., food, shelter, clothing) and host events to build community, which increases social inclusion and improves mental health. As such, PEs found it easier to motivate clients with religious beliefs to re-engage in care.

#### Social network-level facilitators

Emergent social network-level themes included trusted community recommendations, patient navigation services, commitment to family and children, and employer support. Trusted community recommendations and positive clinic reputations were seen as essential to promoting healthcare services to clients, including HIV, HCV and OST services. PEs characterized communication networks within PWID communities that shared both positive and negative clinical experiences, which often impacted client engagement or re-engagement in care. When discussing HCV services, PEs stressed the importance of considering community perceptions and clinic reputations when developing HCV care delivery strategies.

At the center of client care was the unanticipated theme of patent navigation services carried out by PEs providing the highest level of healthcare support and coordination, which often falls outside of the PE job description and without additional compensation. Specifically, PEs described (a) tracking and reminding clients of medical appointments, (2) absorbing clients’ medical transportation costs, (3) navigating administrative processes, (4) mediating patient-provider discussions and (5) providing treatment adherence reminders. Hawi recalls neglecting her medical appointments at the height of her addiction, which motivates her persistent follow-up with clients:

*I go with them to the hospital and when they have been given medication, I ensure they get them [medication], and if they are given a follow-up [appointment] I accompany them to the hospital again until they are done.* Hawi, F, Age 34 yrs, PE for 3 years

Ultimately, PEs described additional hours in providing ancillary services to clients that are integral to HIV/HCV care and achieving viral suppression; however, these services remain largely uncompensated and unrecognized by the medical community.

In addition, men and women discussed slightly different motivations for engaging or re-engaging in care. For women, commitment to family and children often motivates PEs and their female clients to follow medical recommendations. For men, influential community members like religious figures, PE colleagues and supportive employers provides motivation to remain or re-engage in HIV/HCV services. Moha (M, age 34 years) described the positive impact his former employer had on returning to care. Moha’s employer permitted him to take time-off to attend medical appointments and encouraged him to take medical leave to address his substance use disorder, while maintaining job security. Both social networks (i.e., PWID, family, children, and employers) and patient navigation services provided by PEs are instrumental in facilitating clients’ engagement in care.

#### Community-level facilitators

High-quality service delivery, harm reduction services, and peer educator programs emerged as community-level themes and promoters of HIV/HCV care. All PEs credited access to harm reduction organizations, like SAPTA, for their improved physical and mental health. PEs relate to clients’ initial skepticism of harm reduction agencies, but share their positive experiences with non-judgmental staff, access to social services (e.g., laundry, two meals per day, and shower facilities) and quality healthcare services within harm reduction facilities. Wairimu's (F, age 27 years) initial motivation for visiting SAPTA was to access social services, which created a level of trust and later transitioned into HIV, HCV and OST service engagement. More often than not, PEs credited harm reduction facilities as mediators or “bridges” between clients and high-quality HIV/HCV care.

Harm reduction programs apply grassroots service delivery principles through the PE program, training PEs, “*to meet people where they are*”, and provide harm reduction services in dens where clients reside. These activities establish rapport between PEs and clients, with PEs drawing on their lived experiences and serving as positive role models. Eventually, PEs are able to broach the topic of HIV/HCV prevention, testing and care. Martin (M, age 50 years) explains that several of his clients are, “*feeling like it’s the end of the world... but when they look at people like us [PEs], they get hope.*” Employing PEs is a critical step in bridging the gap between PWID and medical communities that ultimately improves access to HIV, HCV and ancillary medical services.

#### Policy-level facilitators

PEs described two overarching policy-level themes, which included: supportive harm reduction and OST service policies and integrating medicalized approaches within law enforcement agencies. Three PEs provided historical contexts prior to the advent of harm reduction programs. Martin (M, age 50 years) shared injection equipment and engaged in “flashblood,” where he would inject another peer’s blood to avoid withdrawal symptoms, which is how he believes he contracted HIV and HCV. Following the national support of harm reduction and OST programs, PEs noted the subsequent uptake of HIV/HCV prevention and care among PWID. Muniu (M, age 39 years) engaged in heroin and benzodiazepine use for more than 10-years and posits when “*methadone came [to Kenya], life began*.” Until harm reduction and OST programs were nationally recognized public health strategies, PEs found it extremely difficult to assist clients in accessing HIV/HCV services.

Until recently, PEs noted that punitive punishments for drug possession were the standard practice; however, more recently harm reduction programs have partnered with law enforcement agencies to provide education around medicalized approaches to assist PWID. PEs described three accounts of law enforcement agents taking a medicalized approach by encouraging clients to pursue OST when they were found in possession of opioids and/or drug equipment. In these instances, law enforcement agents provided clients with two options: jail or OST enrollment. While PEs felt medicalized approaches were inconsistently implemented by law enforcement agencies, they highlighted the promising effects of law enforcement being a gateway to OST, HIV, HCV and harm reduction services.

### Recommended services

PEs recommended HIV/HCV resources and services that would assist them in providing outreach and harm reduction services, which were categorized by: social, medical and ancillary services. Within the requested social services, PEs advocated for occupational and vocational training programs for clients that provide pathways towards financial independence and stable housing options, which would allow them to prioritize their health. Vocational training spanned basket weaving, cosmetology, and typing classes. Four PEs suggested evaluating a client’s education and skill-level to offer opportunities for re-certification or occupational re-integration where appropriate. PEs noted clients with small businesses, mainly shoe-shining and car washing, could benefit from small business loan programs to purchase supplies. Nearly all PEs advocated for occupational programs for clients to increase their financial independence and improve their living conditions by transitioning away from drug environments, so that PEs could better assist with HIV/HCV service engagement.

Nearly all PEs requested integrated medical services, which included HIV, HCV, OST and ancillary services within established harm reduction organizations. PEs described the time, effort and cost of escorting clients to multiple clinics that serve the general population, which produced lapses in care and less time spent conducting outreach. Two PEs suggested that providers and clinical frontline workers should be cross-trained on substance use disorders to reduce stigma and discrimination. PEs that have clients who frequently travel back to their villages, advocated for harm reduction mobile units that provide HIV/HCV services in the villages. Additionally, six PEs requested training in first aid, resuscitation, and basic wound care that they can apply while conducting outreach. PEs are an integral part of HIV/HCV care, yet their training is limited to outreach, harm reduction education and informal patient navigation services, with several PEs requesting additional responsibilities to better serve their clients.

Recommendations for ancillary resources included mental health services, family counseling and re-unification services, national HCV educational campaigns, and non-traditional services like physical activity space to improve *kujithamini*, or self-esteem, and mental health. Twelve PEs stressed the importance of addressing clients’ mental and physical health in parallel to increase their overall wellbeing. Specifically, PEs recommended mental health and counseling services equipped to deal with co-occurring disorders (e.g., trauma, depression, schizophrenia), as well as, family counseling and reunification services. Nearly all PEs described their client’s as having a basic understanding of HIV prevention, but lacked awareness around HCV prevention. PEs advocated for national HCV campaigns to educate community members and low literacy campaigns tailored towards PWID communities. Finally, two PEs suggested providing physical activity space and workout equipment in harm reduction facilities, explaining that improved physical appearance increases a client’s confidence, and ultimately, keeps them engaged in HIV/HCV care. While non-traditional, ancillary programs are not widely promoted, they may offer unique solutions in addressing HIV/HCV care barriers.

## Discussion

This qualitative analysis applies the MSEM as an underlying framework to better understand HIV/HCV barriers and facilitators to care through the unique lens of PEs who offer both lived-experiences as former PWID, and professional experiences of providing outreach services to hidden PWID communities in Nairobi, Kenya. Barriers and facilitators to HIV/HCV care were identified within each level of the MSEM, including individual, social network, community and policy-levels, and were specific to Kenya’s HIV and HCV epidemics among PWID, a key affected population [14]. Notably, stigma was an overarching theme that touched each level of the MSEM and greatly impacted HIV/HCV care. Additional resources and service recommendations to aid PEs included additional medical, social and ancillary support services. Leveraging the unique perspectives of PEs through an ecological framework, posits intervention strategies that address one level of the MSEM may have limited effectiveness in improving HIV/HCV care. Moreover, HIV/HCV interventions should be developed by multiple stakeholders including current PWID, PEs, medical professionals, law enforcement agents, and policy-makers.

Addressing policy-level barriers through a top-down approach may address multiple HIV/HCV-related barriers at various levels of the MSEM with suggestions to (a) expand integrated services beyond the current OST, HIV, and HCV services; (b) increase OST capacity and treatment options; and (c) advocate for drug policies that emphasize medicalized, rather than punitive approaches. Several studies point to the benefits of patient-centered, integrated care models that provide HIV, HCV, OST and ancillary services within trusted harm reduction organizations and employ medical staff trained on the specific needs of PWID [29, 30]. Globally, integrated approaches have effectively improved health outcomes among PWID, including increased community viral suppression, and were more cost-effective compared to decentralized care models [30–32]. In addition, establishing integrated services and policies for PWID care can directly impact community and individual-level barriers by lowering transportation costs and reducing the risk of experiencing withdrawal symptoms while commuting to clinical appointments. However, PEs and PWID have expressed confidentiality concerns attributed to clinical infrastructure, in our study and elsewhere, which should be addressed [33]. More recently, harm reduction facilities have incorporated additional medical services (e.g., ART, physical exams, tuberculous testing), which may provide a foundation for integrating HCV care in the near future. As such, integrated services within trusted harm reduction facilities offer a high level of patient-centered care and the potential to increase HIV/HCV service uptake among PWID.

To address logistical and financial barriers, including mobility to and from villages, OST services should consider incorporating more convenient treatment options like take-home buprenorphine, which has been associated with improved HIV/HCV care outcomes [34, 35]. Currently, daily observed methadone is Kenya’s standard of care for OST; however, service coverage is limited and requires patients to attend daily clinic appointments which presents time, financial and logistic barriers for PWID [17, 20]. Early concerns pointed to the effectiveness, misuse and increased overdose risks associated with take-home buprenorphine treatment; however, new evidence highlights the multiple benefits, including increased HIV/HCV treatment initiation and adherence, negative opioid urinalysis, HIV viral suppression and HCV viral clearance among PWID [34–36]. Currently, Kenya’s Essential Medicines List (2019) includes methadone and buprenorphine as approved OST; however, funding constraints limit methadone service access and buprenorphine access is scarce [37]. Thus, efforts to prioritize OST options and increase availability may address opioid use disorders and multiple HIV/HCV barriers.

Outside of healthcare services, punitive drug laws and harm reduction policies further marginalize PWID communities and often disrupted HIV, HCV and OST care. Recent OST service modifications include less punitive, and more strengths-based approaches following a positive urinalysis for opioids (e.g., counselling, goal setting) to meet PWID where they are in their addiction [14, 38]. After the completion of our qualitative study, SAPTA and other harm reduction facilities partnered with local law enforcement agencies to provide education on medicalized approaches when working with PWID. Currently, a few local police jurisdictions offer PWID the option of drug treatment or a jail sentence upon arrest; however, constraints exist on OST capacity and discriminatory actions carried out by law enforcement agents with the perception that substance use is a moral choice, rather than a mental health issue. Global funding agencies and policy makers should prioritize (a) integrating OST, HIV, and HCV services within trusted harm reduction facilities, (b) increasing capacity and disseminating OST options, including buprenorphine, and (c) advocating for local and national policies that adapt medicalized approaches when working with PWID.

With the anticipated release of DAAs in 2021, HCV prevention, testing and care services are not well understood by PWID communities, supporting the need for educational awareness campaigns. Until recently, HCV treatment in Kenya was limited to interferon-based options, which are cost prohibitive, produced multiple side effects and were largely ineffective (< 50% viral clearance) [1]. According to the WHO, DAAs are the standard of care for HCV due to their convivence and increased effectiveness [39, 40]. Similarly, educational campaigns targeting PWID could incorporate messaging that informs PWID of advanced HIV/HCV disease symptoms, which can be mistaken as withdrawal symptoms, and highlight the benefits of HIV/HCV engagement in care and treatment adherence. Furthermore, targeted HCV educational campaigns for medical providers, PWID, peer educators and other harm reduction specialists are fundamental to HCV elimination, which is largely achievable in the DAA era.

Finally, addressing the mutually reinforcing effects of intra- and interpersonal stigma (e.g., social network, community and policy level) at multiple levels of the MSEM can greatly improve the physical and mental wellbeing of PWID communities. Policy-level recommendations include broad communication efforts enforcing Kenya’s National AIDS and STI Control Programme Guidelines that condemn community violence as a form of retaliation and moral cleansing, paired with healthcare provider trainings emphasizing harm reduction approaches [41]. Alternatively, political ambivalence towards community violence will continue to reinforce issues of stigma towards PWID across all levels of the MSEM and should be prioritized. At the social network-level, cognitive behavioral interventions combined with family and couple counselling have noted promising results in reducing stigma [42]. Intrapersonal interventions include personalized counseling sessions on “coping” with experienced stigma or changing personal environments, which when well-timed and supported by sufficient resources have been effective [43]. While the MSEM maintains distinct levels, pragmatically, these levels are interconnected. Thus, addressing stigma at one level of the MSEM may reduce stigma within the other levels, with policy- and community-level interventions being able to change socio-cultural norms that can directly affect interpersonal beliefs. Stigma against injection drug use carries important implications for PWID health, and currently, there are limited evidence-based interventions that reduce HIV/HCV and/or substance use-related stigma in Kenya, highlighting opportunities to adapt, develop and evaluate strategies in the future [43, 44].

### Limitations

These findings reflect the personal and professional experiences of PEs from two harm reduction facilities in Nairobi, Kenya, and may not be representative of all PEs or PWID experiences. Additional insight may be gained through the experiences of providers, local policy makers, and persons who are actively injecting drugs at the time of the interview. Second, the interview setting included PEs’ place of employment, which may have induced social desirability bias. However, this was felt to be minimal as several PEs were comfortable discussing sensitive topics like illegal activities, instances of relapse, and negative employment experiences, including low salaries, low transportation reimbursement, and personal cell phone use. Thirdly, this study took place prior to the release of DAAs, with the anticipation that information gained from this work could inform HCV program planning.

## Conclusion

Applying the MSEM as a guiding framework and drawing upon the experiences of PEs to discuss personal and professional HIV/HCV barriers and facilitators to care within Nairobi, Kenya, offers a grassroots perspective to improving HIV/HCV care among PWID that should be considered when designing future initiatives. Moreover, addressing barriers and supporting existing facilitators at multiple levels of the MSEM may offer more effective approaches to increasing HIV/HCV care uptake. Recommendations largely focused on policy and community-level interventions to supplement existing services and awareness around issues that affect PWID, with the ultimate goal of achieving community HIV viral suppression and HCV viral clearance among PWID in Kenya.

## Data Availability

Study materials and data that support the findings of this study are available from the corresponding author (NLB) on reasonable request. The data are not publicly available due them containing information that could compromise research participant privacy/consent.

## Abbreviations

AIDS: Acquired immunodeficiency syndrome
ART: Antiretroviral treatment (HIV treatment)
DAA: Direct acting antivirals (hepatitis C treatment)
HIV: Human immunodeficiency virus
HCV: Hepatitis C virus
MSEM: Modified social ecological model
NSP: Needle/syringe programs
PE: Peer educator
PWID: People who inject drugs
OST: Opioid substitution therapy
SAPTA: Support for addictions prevention and treatment in Africa (harm reduction organization)
STI: Sexually transmitted infection
WHO: World Health Organization

## References

[1] Akiyama MJ, Cleland CM, Lizcano JA, Cherutich P, Kurth AE (2019). Prevalence, estimated incidence, risk behaviours, and genotypic distribution of hepatitis C virus among people who inject drugs accessing harm-reduction services in Kenya: a retrospective cohort study. Lancet Infect Dis.

[2] Degenhardt L, Peacock A, Colledge S, Leung J, Grebely J, Vickerman P, Stone J, Cunningham EB, Trickey A, Dumchev K, Lynskey M (2017). Global prevalence of injecting drug use and sociodemographic characteristics and prevalence of HIV, HBV, and HCV in people who inject drugs: a multistage systematic review. Lancet Glob Health.

[3] Kurth AE, Cleland CM, Des Jarlais DC, Musyoki H, Lizcano JA, Chhun N, Cherutich P (2015). HIV prevalence, estimated incidence, and risk behaviors among people who inject drugs in Kenya. J Acquir Immune Defic Syndr.

[4] National AIDS and STI Control Programme (NASCOP). 2010–2011 Integrated biological and behavioural surveillance survey among key populations in Nairobi and Kisumu, Kenya. Nairobi: Government of Kenya, Ministry of Public Health and Sanitation; 2014 Nov. Cited 2021 July 10. https://globalhealthsciences.ucsf.edu/sites/globalhealthsciences.ucsf.edu/files/pub/final_report_keypops_ibbs_nov_24_2014_print.pdf

[5] National AIDS Control Council (NACC), Kenya Ministry of Health. Kenya HIV estimates report 2018. Nairobi, Kenya, 2018 Oct. Cited 2021 July 10. https://nacc.or.ke/wp-content/uploads/2018/11/HIV-estimates-report-Kenya-20182.pdf

[6] Tun W, Sheehy M, Broz D, Okal J, Muraguri N, Raymond HF, Musyoki H, Kim AA, Muthui M, Geibel S (2015). HIV and STI prevalence and injection behaviors among people who inject drugs in Nairobi: results from a 2011 bio-behavioral study using respondent-driven sampling. AIDS Behav.

[7] Muriuki BM, Gicheru MM, Wachira D, Nyamache AK, Khamadi SA (2013). Prevalence of hepatitis B and C viral co-infections among HIV-1 infected individuals in Nairobi. Kenya BMC Res Notes.

[8] Operskalski EA, Kovacs A (2011). HIV/HCV co-infection: pathogenesis, clinical complications, treatment, and new therapeutic technologies. Curr HIV-AIDS Rep.

[9] Musyoki H, Bhattacharjee P, Sabin K, Ngoksin E, Wheeler T, Dallabetta G (2021). A decade and beyond: learnings from HIV programming with underserved and marginalized key populations in Kenya. J Int AIDS Soc.

[10] Smith-Palmer J, Cerri K, Valentine W (2015). Achieving sustained virologic response in hepatitis C: a systematic review of the clinical, economic and quality of life benefits. BMC Infect Dis.

[11] Rich KM, Bia J, Altice FL, Feinberg J (2018). Integrated models of care for individuals with opioid use disorder: how do we prevent HIV and HCV?. Curr HIV/AIDS Rep.

[12] Morgan JR, Servidone M, Easterbrook P, Linas BP (2017). Economic evaluation of HCV testing approaches in low and middle income countries. BMC Infect Dis.

[13] Akiyama MJ, Muller A, Huang O, Lizcano J, Nyakowa M, Riback L, Ross J, Bundi H, Kulabi ES, Mwangi AM, Musyoki H (2021). Hepatitis C-related knowledge, attitudes and perceived risk behaviours among people who inject drugs in Kenya: a qualitative study. Glob Pub Health.

[14] Abdool R (2016). Policy change towards implementing harm reduction in Sub-Saharan Africa. Int J Drug Policy.

[15] National AIDS and STI Control Programme (NASCOP), Kenya Ministry of Health. Standard operating procedures for establishing and operating drip-in centres for key populations in Kenya, November 2016. Cited 2021 July 10. Available from: https://www.phdaf.org/wp-content/uploads/2021/03/DIC-SOP_PRINT.pdf

[16] National AIDS and STI Control Programme (NASCOP), Kenya Ministry of Health. The national guidelines for HIV/STI programming with key populations. Nairobi, Kenya, 2014 October. Cited 2021 July 10. https://www.icop.or.ke/wp-content/uploads/2016/10/KP-National-Guidelines-2014-NASCOP.pdf

[17] Stone J, Fraser H, Walker JG, Mafirakureva N, Mundia B, Cleland C, Kigen B, Musyoki H, Waruiru W, Ragi A, Bhattacharjee P. Modelling the impact of prevention and treatment interventions on HIV and hepatitis C virus transmission among people who inject drugs in Kenya. medRxiv. 2021. 10.1097/QAD.0000000000003382 PMC9671825 36111533

[18] Kenya Ministry of Health. The national protocol for treatment of substance use disorders in Kenya. Mental Health & Substance Abuse Management Unit, Nairobi, Kenya, 2017. Cited 2021 July 10. https://www.afro.who.int/sites/default/files/2017-09/The%20National%20Protocol%20for%20treatments%2014%2007%202017.pdf

[19] Guise A, Rhodes T, Ndimbii J, Ayon S, Nnaji O (2016). Access to HIV treatment and care for people who inject drugs in Kenya: a short report. AIDS Care.

[20] Larney S, Peacock A, Leung J, Colledge S, Hickman M, Vickerman P, Grebely J, Dumchev KV, Griffiths P, Hines L, Cunningham EB (2017). Global, regional, and country-level coverage of interventions to prevent and manage HIV and hepatitis C among people who inject drugs: a systematic review. Lancet Glob Health.

[21] National AIDS and STI Control Programme (NASCOP), Kenya Ministry of Health. Manual for training peer educators for programs with people who inject drugs. Nairobi, Kenya, 2017 June. Cited on 2021 July 10. Available from: https://hivpreventioncoalition.unaids.org/wp-content/uploads/2019/01/NASCOP2017_Manual-for-Training-Peer-Educators-for-Programs-with-Female-Sex-Workers-Participants-Handbook_Kenya.pdf

[22] Baral S, Logie CH, Grosso A, Wirtz AL, Beyrer C (2013). Modified social ecological model: a tool to guide the assessment of the risks and risk contexts of HIV epidemics. BMC Pub Health.

[23] Larios SE, Lozada R, Strathdee SA, Semple SJ, Roesch S, Staines H, Orozovich P, Fraga M, Amaro H, de la Torre A, Magis-Rodriguez C (2009). An exploration of contextual factors that influence HIV risk in female sex workers in Mexico: the Social Ecological Model applied to HIV risk behaviors. AIDS Care.

[24] El-Bassel N, Strathdee SA (2015). Women who use or inject drugs: an action agenda for women-specific, multilevel and combination HIV prevention and research. J Acquir Immune Defic Syndr (1999).

[25] Mburu G, Limmer M, Holland P (2019). HIV risk behaviours among women who inject drugs in coastal Kenya: findings from secondary analysis of qualitative data. Harm Reduct J.

[26] Braun V, Clarke V (2006). Using thematic analysis in psychology. Qual Res Psychol.

[27] Patton MQ (2014). Qualitative research & evaluation methods: integrating theory and practice.

[28] Fereday J, Muir-Cochrane E (2006). Demonstrating rigor using thematic analysis: a hybrid approach of inductive and deductive coding and theme development. Int J Qual Methods.

[29] Belani H, Chorba T, Fletcher F, Hennessey K, Kroeger K, Lansky A, Leichliter J, Lentine D, Mital S, Needle R, O’Connor K (2012). Integrated prevention services for HIV infection, viral hepatitis, sexually transmitted diseases, and tuberculosis for persons who use drugs illicitly: summary guidance from CDC and the US Department of Health and Human Services. MMWR.

[30] Haldane V, Cervero-Liceras F, Chuah FL, Ong SE, Murphy G, Sigfrid L, Watt N, Balabanova D, Hogarth S, Maimaris W, Buse K (2017). Integrating HIV and substance use services: a systematic review. J Int AIDS Soc.

[31] Bachireddy C, Soule MC, Izenberg JM, Dvoryak S, Dumchev K, Altice FL (2014). Integration of health services improves multiple healthcare outcomes among HIV-infected people who inject drugs in Ukraine. Drug Alcohol Depend.

[32] Tran OC, Bruce RD, Masao F, Ubuguyu O, Sabuni N, Mbwambo J, Lambdin BH (2015). Implementation and operational research: linkage to care among methadone clients living with HIV in Dar es Salaam, Tanzania. J Acquir Immune Defic Syndr.

[33] Guise A, Ndimbii J, Igonya EK, Owiti F, Strathdee SA, Rhodes T (2019). Integrated and differentiated methadone and HIV care for people who use drugs: a qualitative study in Kenya with implications for implementation science. Health Policy Plan.

[34] Norton BL, Beitin A, Glenn M, DeLuca J, Litwin AH, Cunningham CO (2017). Retention in buprenorphine treatment is associated with improved HCV care outcomes. J Subst Abuse Treat.

[35] Springer SA, Qiu J, Saber-Tehrani AS, Altice FL (2012). Retention on buprenorphine is associated with high levels of maximal viral suppression among HIV-infected opioid dependent released prisoners. PLoS ONE.

[36] Altice FL, Bruce RD, Lucas GM, Lum PJ, Korthuis PT, Flanigan TP, Cunningham CO, Sullivan LE, Vergara-Rodriguez P, Fiellin DA, Cajina A (2011). HIV treatment outcomes among HIV-infected, opioid-dependent patients receiving buprenorphine/naloxone treatment within HIV clinical care settings: results from a multisite study. J Acquir Immune Defic Syndr.

[37] Kenya Ministry of Health. Kenya essential medicines list 2019. Nairobi, Kenya, 2019 November. Cited on 2021 July 10. https://www.health.go.ke/wp-content/uploads/2020/03/Kenya-Essential-Medicines-List-2019.pdf

[38] Craw JA, Gardner LI, Marks G, Rapp RC, Bosshart J, Duffus WA, Rossman A, Coughlin SL, Gruber D, Safford LA, Overton J (2008). Brief strengths-based case management promotes entry into HIV medical care: results of the antiretroviral treatment access study-II. J Acquir Immune Defic Syndr.

[39] Boglione L, Mornese Pinna S, De Nicolo A, Cusato J, Cariti G, Di Perri G, D'Avolio A (2017). Treatment with direct-acting antiviral agents of hepatitis C virus infection in injecting drug users: a prospective study. J Viral Hepat.

[40] World Health Organization (WHO). Guidelines for the care and treatment of persons diagnosed with chronic hepatitis C virus infection. Geenva, Switzerland, 2018 July. Cited on 2021 May 10. http://apps.who.int/iris/bitstream/handle/10665/273174/9789241550345-eng.pdf 30307724

[41] National AIDS and STI Control Programme (NASCOP). National key populations communication strategy 2014–2017: Communication strategy for sex workers, people who inject drugs and men who have sex with men. Nairobi, Kenya, 2014 November. Cited on 2021 July 20. http://icop.or.ke/wp-content/uploads/2016/10/KP-Communication-Strategy-2014_2017.pdf

[42] McHugh RK, Hearon BA, Otto MW (2010). Cognitive behavioral therapy for substance use disorders. Psychiatric Clin.

[43] Cook JE, Purdie-Vaughns V, Meyer IH, Busch JT (2014). Intervening within and across levels: a multilevel approach to stigma and public health. Soc Sci Med.

[44] Sengupta S, Banks B, Jonas D, Miles MS, Smith GC (2011). HIV interventions to reduce HIV/AIDS stigma: a systematic review. AIDS Behav.

